# Navigating adulthood with PKU: metabolic outcomes, quality of life, and mental health 4.5 years post-transition

**DOI:** 10.1186/s13023-025-04186-1

**Published:** 2026-01-09

**Authors:** Antonia Albers, Nadine Kuniß, Christof Kloos, Gunter Wolf, Sebastian Schmidt, Nicolle Müller

**Affiliations:** 1 Endocrinology and Metabolic Diseases, Department of Internal Medicine III, University Hospital Jena, Am Klinikum 1, 07747 Jena, Germany; 2 Institute of Agriculture and Nutritional Science, Martin-Luther-Universität, 06120 Halle, Germany; 3 MED:ON Academy, Jena, Germany

**Keywords:** Transition, PKU, Quality of life, Psychiatric comorbidities, Adults, Adherence

## Abstract

**Introduction:**

Adult care for individuals with phenylketonuria (PKU) remains limited, as many centers prioritize pediatric patients. Structured transition into adult care is essential to maintain metabolic stability, adherence, and monitor comorbidities - particularly psychiatric ones. This study evaluates metabolic control, quality of life (QoL), and the impact of psychiatric comorbidities in adults with PKU after transition.

**Methods:**

This retrospective study included 45 adults with PKU assessed 4.5 years after entering adult care. Data from transition and annual follow-ups were analyzed. Phenylalanine (Phe) concentrations were assessed using venous blood samples and dried blood spots. Dietary habits and amino acid mixtures (AAM) intake were recorded via interviews. QoL was measured with the *PKU-QOL* (0-100; lower scores indicate better QoL) and WHO-5 (0–25, higher scores indicate better well-being). Comorbidities were obtained from medical records.

**Results:**

Mean Phe concentration remained stable (998.3 ± 290.4µmol/l). Adherence to low-protein diet increased from 77.8% to 100%, and AAM intake from 62.5% to 85.8% over 4.5 years. Consistent AAM use (≥ 3x/day) and adherence to low-protein diet were each associated with lower Phe levels compared to no AAM or no diet (− 362.9µmol/l and − 304.1µmol/l, *p* < 0.05). Overall, 89% had comorbidities, most commonly psychiatric disorders (31%). These individuals showed higher Phe levels (1169µmol/l vs. 823µmol/l, *p* < 0.05), lower dietary adherence (33% vs. 70%, *p* < 0.01), and greater QoL impairment.

**Conclusion:**

Structured transition into adult care supports metabolic stability. However, psychiatric comorbidities are strongly linked to poorer adherence and worse metabolic and psychosocial outcomes. Integrating psychological support into adult PKU care is therefore essential.

**Synopsis:**

A structured transition into adult care can support the maintenance of metabolic control and quality of life in adults with PKU, especially given the high prevalence of psychiatric comorbidities, which can negatively affect adherence.

**Supplementary Information:**

The online version contains supplementary material available at 10.1186/s13023-025-04186-1.

## Introduction

Phenylketonuria (PKU) is a rare inherited metabolic disorder caused by a genetic defect in phenylalanine hydroxylase (PAH), the enzyme responsible for converting phenylalanine (Phe) to tyrosine [1]. As a result, Phe accumulates in the blood at toxic levels, leading to neurotoxicity and, if left untreated irreversible neurological impairment, including intellectual disability, microcephaly, and epilepsy [2].

With a global prevalence of approximately 1 in 23,930 and 1 in 10,000 in Europe, PKU is the most common inborn error of metabolism [3]. Since 1969–1970, PKU has been routinely diagnosed through newborn screening in Germany, substantially reducing the number of untreated individuals [1, 4]. Early intervention is crucial, and therapy should begin immediately after diagnosis. Standard treatment includes a strict low-protein/Phe-restricted diet supplemented with amino acid mixtures (AAM) to meet nitrogen requirements. Additionally, some patients may benefit from therapy with Sapropterin, the cofactor of PAH. Patients can also be treated with enzyme replacement therapy with Pegvaliase, which is approved for patients 16 years of age and older [5, 6].

Although lifelong treatment is recommended to prevent neurological complications, most specialized metabolic care centers focus primarily on pediatric patients [2, 7]. This lack of adult care facilities forces many patients to remain in pediatric services or experience gaps in medical supervision, increasing the risk of treatment discontinuation and associated health complications [8, 9]. Transition to adult care is therefore critical, as lifestyle changes in adulthood – such as education, employment, independent living, and family planning – may affect disease management [10]. Dietary adherence tends to decline in adults, and studies have shown that only half of the adults with PKU follow dietary recommendations [11].

Additionally, Phe target ranges have changed over time and still vary between countries [2, 12, 13]. Since 2017, European guidelines have recommended maintaining Phe concentrations below 600 µmol/l for adults, whereas previous thresholds were as high as 1200 µmol/l [12]. For children and women before and during pregnancy, stricter recommendations of 120–360 µmol/l apply [2]. Several studies support the recommendation of ≤ 600 µmol/l [14–17] whereas others emphasize the importance of maintaining stable Phe levels rather than strictly lowering them [18]. Long-term studies supporting strict Phe recommendations for adults are lacking [19], although no evidence suggests that low Phe concentration are harmful [16]. Nevertheless, strict dietary therapy can affect quality of life as well as psychological and nutritional aspects [20–22].

Another important aspect of treating adult PKU patients is that the risk of comorbidities increases with age [23]. Psychological, metabolic, cardiovascular and osteological disorders have been reported in association with PKU, although the exact pathophysiological mechanisms are often unknown [24–26]. Mental comorbidities, especially depression, may be explained by competitive inhibition of amino acid transporters at high Phe concentrations [17]. Large neutral amino acids such as tyrosine and tryptophane are transported less efficiently across the blood-brain barrier, and neurotransmitter such as serotonin and dopamine are therefore synthesized to a lesser extent. With regard to metabolic comorbidities, adult PKU patients face an increased risk of lipid disorders, overweight/obesity, and diabetes mellitus [25, 26]. Another common issue is low bone density, often manifesting as osteopenia or osteoporosis [27]. Cardiovascular diseases, particularly hypertension, are more prevalent in both early- and late-diagnosed PKU patients, although the risk of ischemic heart disease is primarily increased in late-diagnosed individuals [26]. The increased risk of these comorbidities again underlines the importance of transitioning adults with PKU from pediatric institutions to adult care.

Health-related quality of life (HRQOL) has become an increasingly factor in treatment success and overall well-being [28, 29]. Questionnaires assessing HRQOL indicate that early-treated PKU patients have HRQOL comparable to that of healthy control groups [24, 30, 31]. However, other studies have demonstrated reduced HRQOL, particularly in specific domains such as cognition, anger, and depressive symptoms, compared with healthy controls. Factors influencing HRQOL, including age, disease severity, and treatment modality, have been identified, although their impact differs substantially across studies [1]. Furthermore, different questionnaires have been used to measure quality of life, which complicates comparisons between studies. While generic measures allow cross-population comparisons, disease-specific instruments provide a more detailed assessment of patient-specific concerns and challenges [32]. To address practical, psychological and social aspects in a health-related manner, the *Phenylketonuria – Quality of Life* (*PKU-QOL*) questionnaire was developed for PKU patients [20]. It is available in six different languages and can be used for children, adolescents, adults, and parents. Current European guidelines recommend assessing HRQOL at least annually and suggest using PKU-specific questionnaires at least once in childhood, adolescence and adulthood [2].

The aim of this study was to investigate the metabolic control after transition to the adult metabolic center and to assess whether dietary restrictions and comorbidities, particularly psychiatric comorbidities, are associated with Phe concentrations and quality of life.

## Methods

### Study design

This retrospective, observational, non-interventional analysis included all patients treated at the adult metabolic center Jena who were diagnosed with classical PKU (ICD-10 E70.0), mild PKU or hyperphenylalaninemia (ICD-10 E70.1). Pregnant women with PKU were not included in the analysis as they follow stricter metabolic control and dietary interventions. The study period extended from the establishment of the metabolic center in December 2019 until April 2024. Data were retrieved from the electronic patient record system (EMIL^®^).

### Parameters

#### Details of transition

Transition begins at approximately 15 years of age and aims to gradually increase the involvement of young patients in their own therapy and dietary management. Toward the end of pediatric care, the future adult providers (endocrinologist and dietitian/nutritionist) attend the final neuropediatric appointment to introduce themselves and ensure continuity of care. Before transfer, the neuropediatric team prepares a detailed transition letter summarizing medical history and relevant treatment information to minimize data loss. At approximately 18 years of age, all involved professionals (neuropediatrician, pediatric dietitian, and both adult-care providers) meet jointly with the patient and parents (or supervisor) to facilitate a structured transfer. On the transition day, a comprehensive medical history is obtained at the adult metabolic center, clinical examinations (including electrocardiogram, bone density measurement and ultrasound, if needed) are performed, and nutritional counseling is provided. Questionnaires assessing HRQoL and well-being are handed out.

#### Annual follow-ups

Patients were evaluated annually, beginning either at the time of transition from a pediatric center, from external care providers, or when restarting treatment. Follow-up visits were scheduled once a year and conducted jointly by an endocrinologist and a nutritionist. To assess metabolic control, Phe concentrations were measured four times per year, consisting of one outpatient measurement (annual visit) and three dried blood spots. Additional measurements were obtained if individual clinical situation required closer monitoring.

At each appointment, patients were asked about their individual target ranges to establish a personalized threshold through shared decision-making with the treating physician and dietitian. Based on these personalized targets, the proportion of values within range between follow-ups was calculated. As part of the annual visit, patients reported their dietary habits and intake of AAM during the weeks preceding the appointment. Patients who calculated their daily Phe intake based on the protein content in foods (using an app or written log) and adhered to a strictly Phe-balanced diet were classified as “strictly calculated diet”. Patients who did not calculate intake but estimated their daily protein consumption were categorized as “estimated low-protein diet”. Diets followed only on weekdays, with frequent exceptions, or vegetarian/vegan diets without protein-intake estimation, were classified as “slightly protein-reduced diet”. Patients reporting that they did not to follow a low-protein diet were categorized as “no diet”. For statistical analysis using mixed models, the calculated and estimated low-protein diet were combined to a single category “low-protein diet”. AAM-intake was retrieved as documented during inpatient and outpatient visits. For regression analysis, intake frequency was grouped as ≥ 3 times per day, 1–2 times per day, and < 1 time per day. Confirmed diagnsoses of comorbidities were also retrieved from the electronic patient records; suspected diagnosis were not included.

### PKU-QoL questionnaire

Quality of life has been assessed annually since 2019 using the *PKU-QOL* questionnaire, validated in 2015 [20, 33]. It is freely available for non-funded academic research and provided by *MAPI Research Trust*, Lyon, France [34]. The 2015 German version and the official *PKU-QOL* user manual were used for scoring.

### Structure of adult PKU-QOL questionnaire

The adult version consists of 65 questions, summarized into 35 domains and organized into four modules: *PKU symptoms*, *PKU in general*, *administration of Phe-free protein substitutes* and *dietary protein restrictions*. The recall period is focused primarily refers to the previous seven days, except for the self-rated health status, which reflects overall perceptions. Responses are recorded using a 4- to 6-point Likert scale [33]. A domain score is calculated if at least 70% of its questions are completed. Domains with insufficient responses are excluded from the analysis. Scores are calculated by summing all item scores, dividing by the number of non-missing responses, and multiplying by 25, resulting in a range from 0 to 100. Lower scores indicate a lower impact on HRQOL (e.g. fewer symptoms or better adherence) [20]. Scores ≤ 25 indicate little to no impact; scores 25–50 reflect a moderate impact; scores > 50 and < 75 indicate a substantial impact; and scores ≥ 75 reflect a very strong impact (very severe and/or very frequent symptoms).

### WHO-5 well-being index

The WHO-Five Wellbeing Index (WHO-5) consists of five questions (scored 0 to 5, total score 0–25) assessing general well-being and mental health [35]. It is completed annually during patient visits and serves as a depression-screening tool referring to the two weeks preceding the assessment. Lower scores indicate poorer well-being. A score < 13 suggests a potential risk of depression [36]. The WHO-5 is freely available from the WHO (*Psychiatric Research Unit*, *Mental Health Center North Zealand*, Hillerød, Denmark).

### Statistical analysis

Statistical analyses were performed by *IBM SPSS Statistics* Version 29. Results for non-normally distributed data are presented as median (MD) and interquartile range (IQR), whereas normally distributed data are reported as mean and standard deviation (SD). The significance level was set at α = 0.05 for all tests. For non-normally distributed data, the Mann-Whitney U test was used to evaluate differences in independent metric and ordinal variables. Differences in nominal variables were analyzed using Pearson´s chi-square test. To identify parameters with the greatest impact on Phe concentrations in PKU patients, mixed linear models were applied. Prior to modeling, correlation analyses (Spearman or Pearson chi-square) were performed to assess associations with Phe levels. Only variables showing significant correlations (*p* < 0.05) were included in the mixed-model analyses. Due to the small sample size, a maximum of four to five variables were included per model. To account for sex and age, separate analyses were conducted for all variables.

## Results

### Patient characteristics

All patients treated at the adult metabolic center in Jena between December 2019 and April 2024 were included in the analysis (*n* = 45). The mean age was 32.8 ± 10.4 years (range: 19.0-64.5 years), and 64.4% of the patients were female (Table 1). Forty individuals (88.9%) had classical PKU, four (8.9%) had mild PKU, and one (2.2%) had mild hyperphenylalaninemia. Two patients (4,4%) were late diagnosed, and two additional patients had initiated dietary treatment at the age of 6 month and approximately 7 years, respectively. All remaining patients started strict Phe-restricted diet within days of diagnosis. Five of the 40 patients with classical PKU (11.1%) had intellectual disability. The majority of the cohort was transferred from the pediatric metabolic center Jena (35 of 45 patients; 77.8%), while others sought specialized care to restart dietary treatment or receive preconception or pregnancy-related counseling. During the study period, seven patients (15.6%) were still attending school or university. A total of 36 patients (82.2%) remained continuously under care in the department; three patients (6.7%) moved and continued treatment elsewhere. Three patients missed their most recent appointment, and two patients (4.4%) were lost to follow-up. Across all appointments, mean WHO-5 scores ranged between 16.1 and 16.8 (range: 2.0–25.0).

**Table 1:** Patient characteristics

Parameters	Unit		*N*		[%]	
Sex	Male / female		16 / 29		35,6 / 64,4	
Transferred from pediatric metabolic center Jena	Pediatric center / extern		35 / 10		77,8 / 22,2	
PKU variant	classical / mild / mild hyperphenylalaninemia		40 / 4 / 1		88,9 / 8,9 / 2,2	
Intellectual impairment	No / Yes		40 / 5		88,9 / 11,1	
Sapropterin therapy	No / Yes		41 / 4		93,3 / 6,7	
Enzyme replacement therapy	No / Yes		44 / 1		97,8 / 2,2	
	**n**	**Mean ± SD**		**MD / IQR**		**Min - Max**
WHO 5-Score total	99	16.5 ± 4.8		18.0 /6.0		2.0–25.0
− Transition/initial appointment	44	16.1 ± 4.1		17.0 / 6.0		7.0–24.0
− 1st annual follow-up	24	16.8 ± 5.7		18.0 / 5.0		2.0–25.0
− 2nd annual follow-up	19	16.3 ± 5.3		18.0 / 7.0		3.0–21.0
− 3rd annual follow-up	11	16.6 ± 4.9		18.0 / 6.0		7.0–24.0

Data were retrieved at the initial appointment, WHO 5-Scores were retrieved from all follow-ups

### Metabolic control

**Fig. 1:**
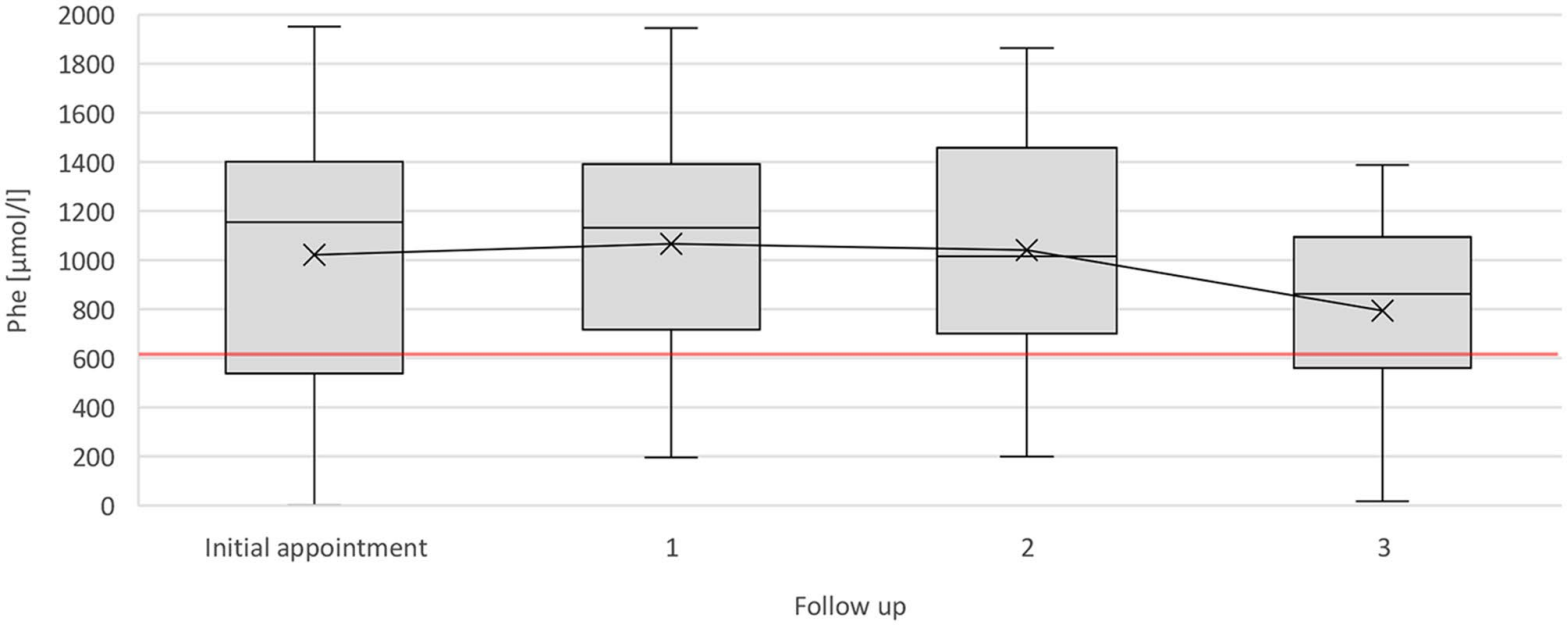
Phe concentration [µmol/l] throughout the appointments. Boxplots with median and mean (x). Red line indicates the European recommendation for Phe concentration (< 600 µmol/l) [2]

Across the observational period, the mean Phe concentration of all patients was 998.3 ± 290.4 µmol/l. At the initial appointment, mean Phe levels were 988.6 ± 469.5 µmol/l (*n* = 44), 1070.2 ± 396.9 µmol/l (*n* = 28) at the first annual follow-up, 1073.3 ± 421.5 µmol/l (*n* = 19) at the second follow-up, and 805.9 ± 392.6 µmol/l (*n* = 13) at the third follow-up (Fig. 1). No significant differences were observed between follow-ups. Most of the patients selected an upper individual Phe target of approximately 900 µmol/l (median values: 780 µmol/l at the initial and first follow-up, 900 µmol/l at the second and third follow-up). Despite stable overall Phe concentrations, the proportion of measurements within each patient´s individual target range declined from 38% (*n* = 43) between initial and first follow-up to 31% (*n* = 27) between the first and second follow-up, and further to 20% (*n* = 20) between the second and third follow-up. The highest proportion in-range was recorded after the third follow-up with 50% (*n* = 12).

### Dietary habits

At the initial appointment, 77.8% of the patients (35/45) adhered to a low-protein diet. Of these, 9 (20.0%) followed a strictly calculated low-protein diet, 16 patients (35.6%) estimated their protein intake, and 10 (22.2%) followed a slightly protein-reduced diet. A total of 22.2% (10/45) reported no dietary restrictions. Dietary adherence improved over time, and by the third annual follow-up all patients adhered to at least a slightly protein-reduced diet. Moreover, a greater proportion followed a strict protein-restricted diet (33.3%, 4/12). In the *PKU-QOL* questionnaire, patients reported that *food temptation*, the *practical impact of dietary protein restriction* and feelings of *guilt if dietary protein restriction was not followed* had the greatest impact on quality of life (median scores between 25.0 and 50.0), indicating a moderate influence. Other aspects of dietary protein restriction had little to no perceived impact (MD ≤ 25).

Regarding AAM intake, 62.5% of patients initially reported consuming AAM at least twice daily, 6.7% once daily, 2.2% a few times per week, and 28.9% did not at all. Over the course of treatment, adherence improved, with 85.8% taking AAM at least twice daily and 50.0% taking it three times per day. At the third annual follow-up, all patients were taking AAM regularly. *Guilt due to poor adherence* and dissatisfaction with *taste of AAM* had the greatest impact on quality of life (mean scores 25.0–50.0), indicating moderate influence; other aspects of the AAM-intake showed little to no impact (MD ≤ 25).

**Fig. 2:**
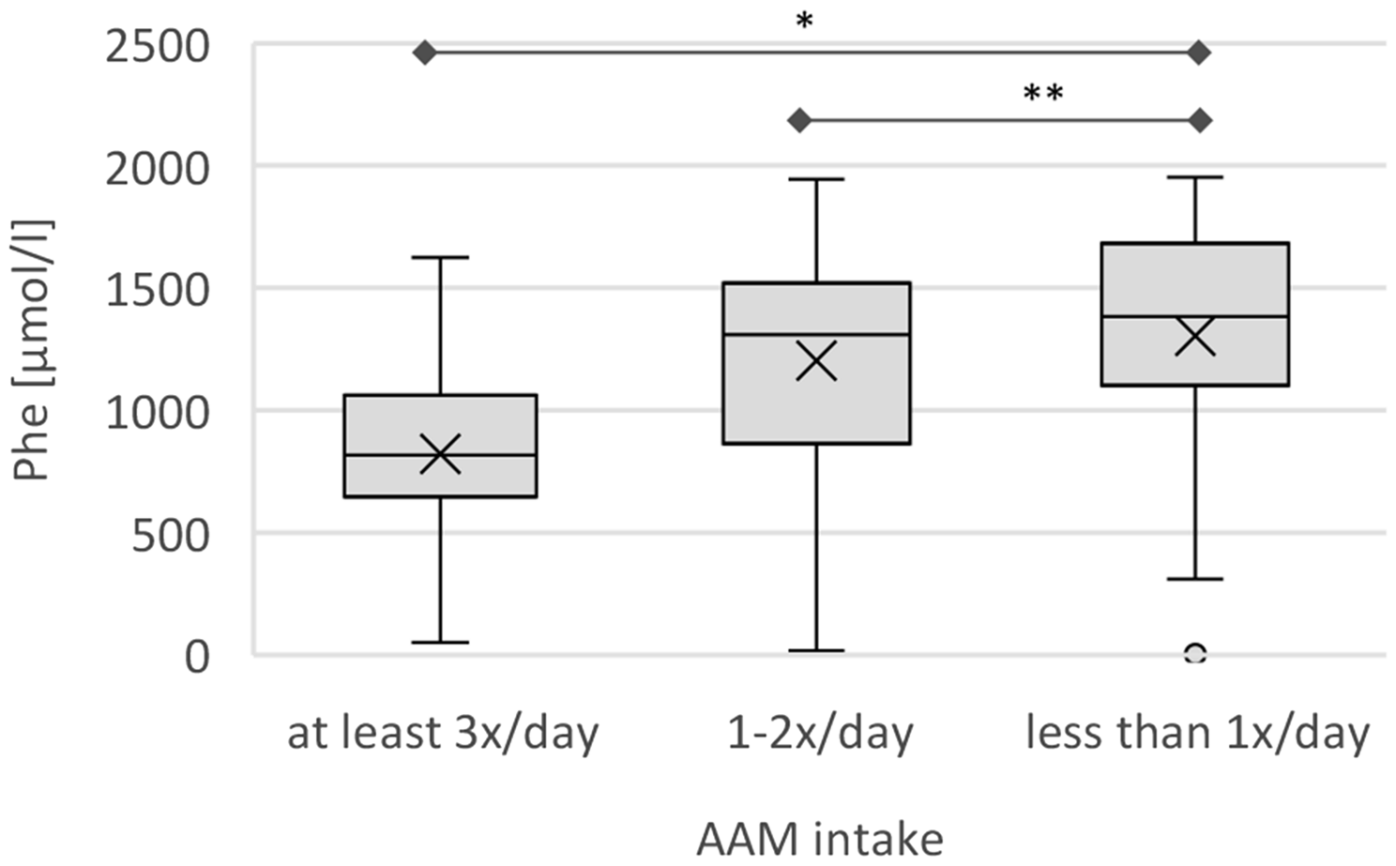
Phe concentration [µmol/l] in association with AAM intake. Phe concentration of all appointments is displayed as boxplots with mean (x) and median (at least 3 times per day, 1- to 2-times per day, less than once per day). Phe: Phenylalanine. * *p* < 0.001, ** *p* < 0.05

**Fig. 3:**
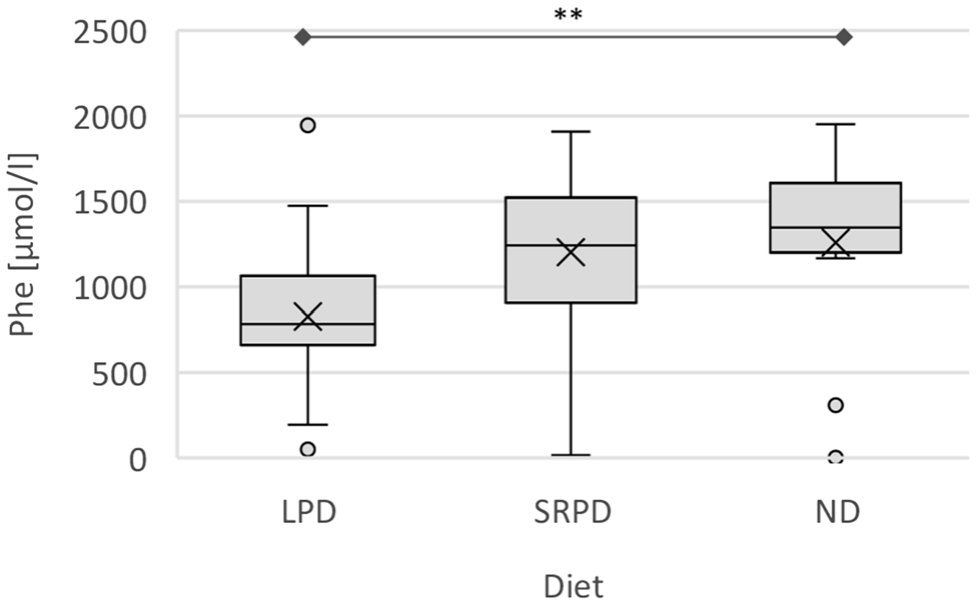
Phe concentrations [µmol/l] in association with dietary habits of adult PKU patients. Phe concentration of all appointments is displayed as boxplots with mean (x) and median. LPD: Low-protein diet; ND: No diet; Phe: Phenylalanine; SRPD: slightly reduced protein diet. ** *p* < 0.05

A significant correlation was observed between the Phe concentrations and both AAM frequency and dietary protein restriction. Patients taking their AAM at least three times per day exhibit a reduction in Phe levels of 362.9 µmol/l (95% CI: 549.9, 175.8; *p* < 0.001) compared to those not taking AAM (Fig. 2). Even an intake of one to two times per day was associated with a reduction of 254.2 µmol/l (95% CI 456.8, 51.7; *p* < 0.05). Similarly, individuals following a calculated or estimated protein-restricted diet demonstrated a reduction in Phe levels of 304.1 µmol/l (95% CI: 568.2, 40.0; *p* < 0.05) compared to those without dietary protein restriction (Fig. 3).

### Comorbidities

**Fig. 4:**
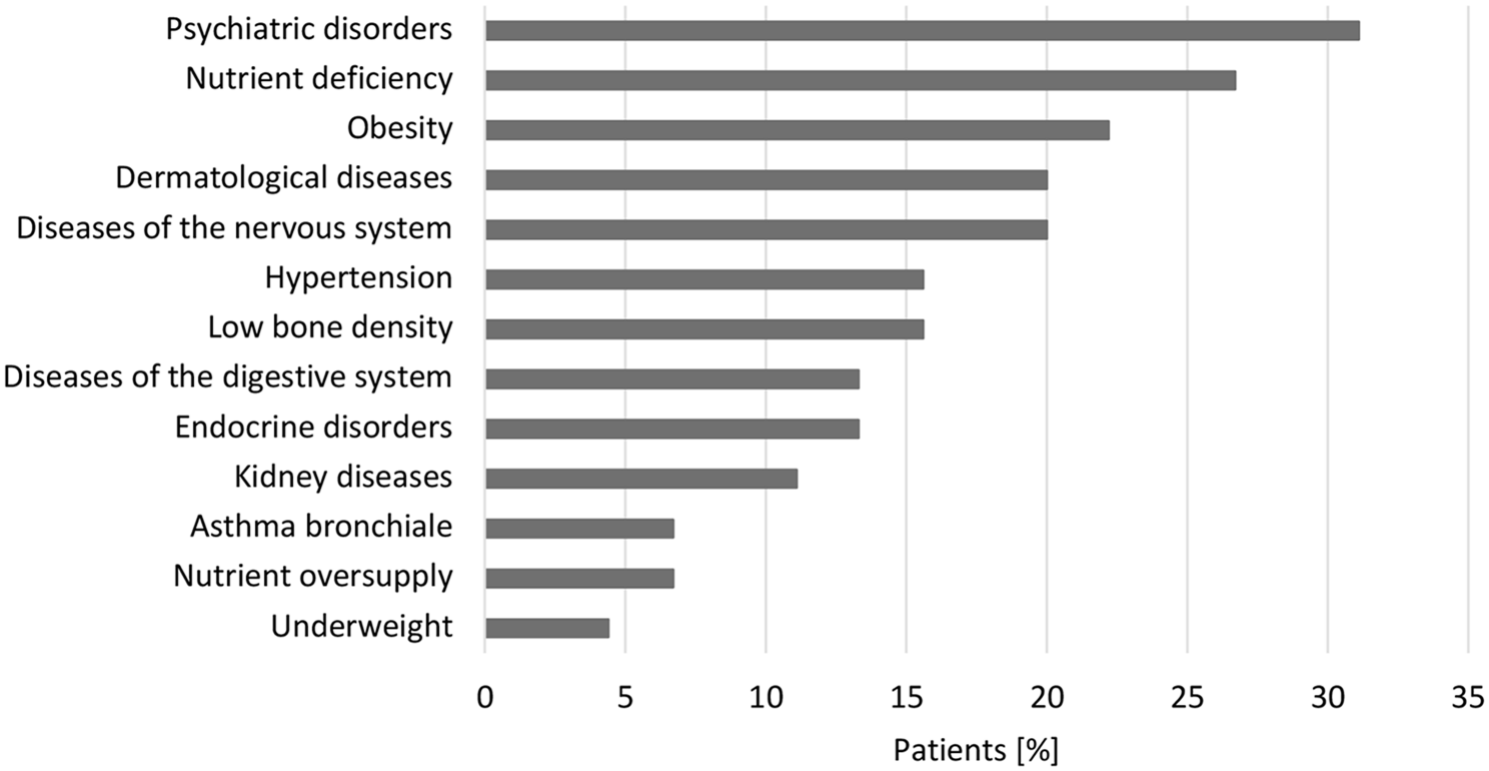
Distribution of comorbidities of adult individuals with PKU by categories in percent

Comorbidities were present in the majority of patients; only 5 of 45 (11.1%) had no documented comorbidity. The most common diagnoses were psychiatric disorders (31.1%), micronutrient deficiencies (26.7%; primarily vitamin D, zinc and iron), obesity (22.2%), skin conditions (20.0%; e.g. eczema, nevi, dermatitis, alopecia) and neurological disorders (20.0%; including migraines, epilepsy) (Fig. 4). Nearly one-third of patients had at least one psychiatric disorder, and eight individuals (17.8%) had multiple psychiatric diagnoses. Anxiety disorders, post-traumatic stress disorders, and hypochondriacal disorders – were diagnosed in six patients (13.3%). Five patients (11.1%) suffered from depression. Less frequent psychiatric conditions included drug addiction, eating disorders and borderline personality disorder (4.4%). Single cases (2.2%) of dementia, paranoid schizophrenia, and attention-deficit/hyperactivity disorder (ADHD) were also documented.

### Comparison of patients with and without psychiatric comorbidities

**Fig. 5:**
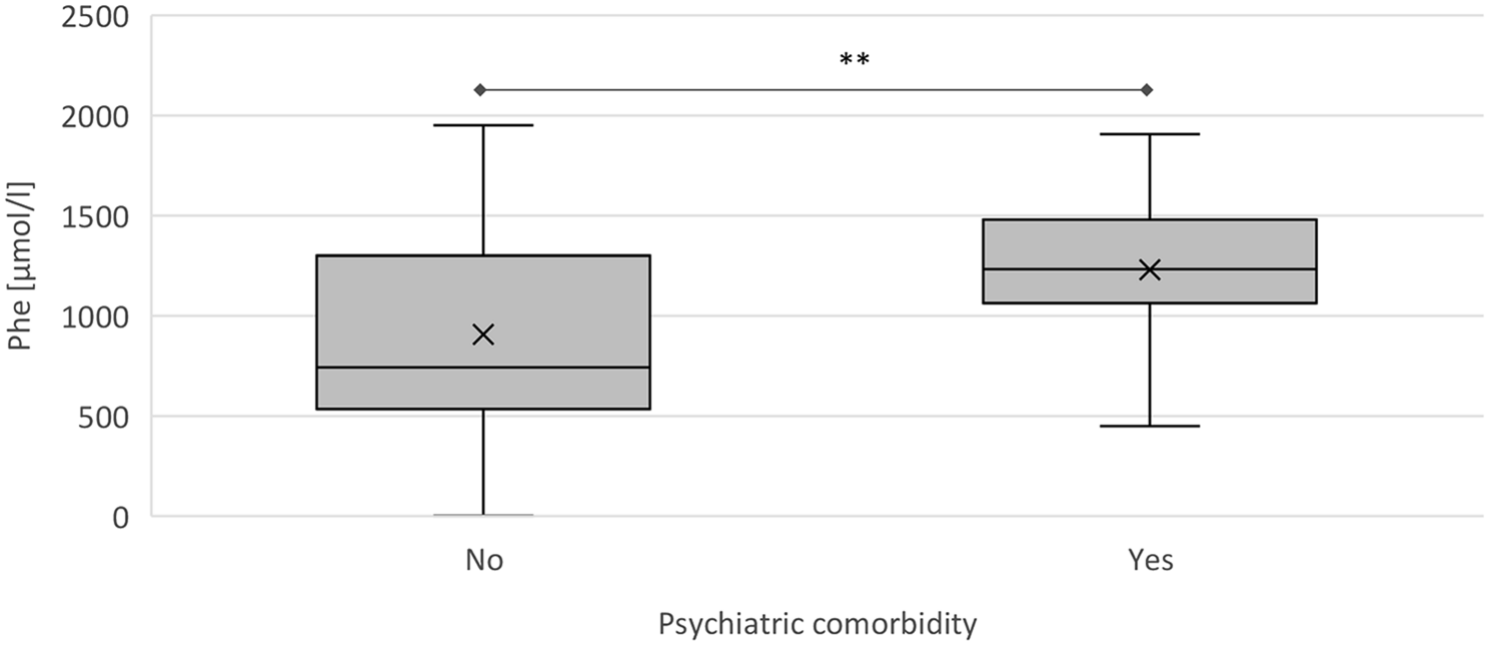
Comparison of Phe concentration [µmol/l] of patients with and without psychiatric comorbidities. Phe concentrations are presented as boxplots with median and mean (x). A mixed model analysis revealed a significant difference of 283.6 µmol/l in Phe concentration (95% CI 561.4, 5.8) between the two groups. ** *p* < 0.05. Phe: phenylalanine

A mixed model analysis revealed a significant association between psychiatric comorbidities and metabolic control. Individuals with psychiatric disorders had significantly higher mean Phe concentrations, with an increase of 283.6 µmol/l (95% CI 561.4, 5.8; *p* < 0.05) compared to those without such comorbidities (Fig. 5). The mean Phe concentration in patients with psychiatric disorders was 1169.8 ± 399.9 µmol/l, compared to 893.0 ± 439.9 µmol/l in those without. Despite similar individualized upper Phe limits (822.8 ± 260.2 µmol/l vs. 768.4 ± 242.0 µmol/l), only 32.5 ± 37.4% of measurements in patients with psychiatric disorders were within target range, compared to 52.2 ± 40.7% in those without (*p* < 0.05).

**Fig. 6:**
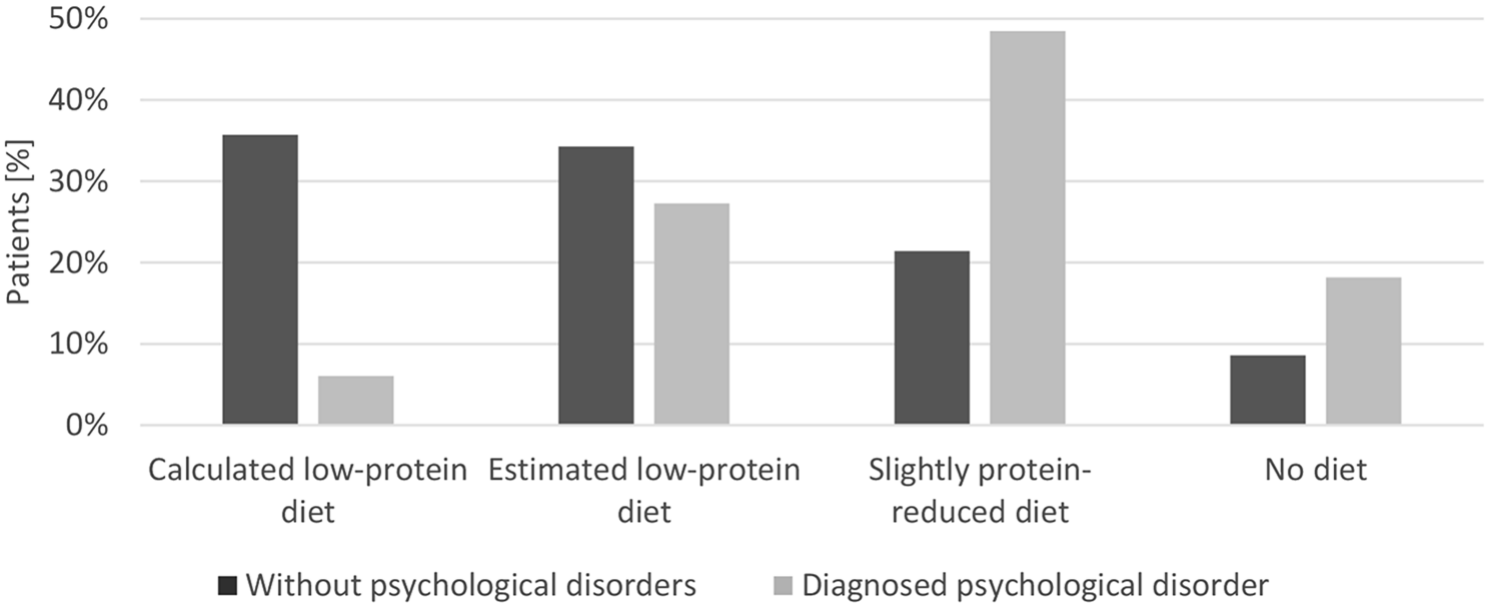
Comparison of dietary habits of patients with and without psychiatric comorbidities. Bars represent the percentage of patients with (light gray) and without (dark gray) diagnosed psychiatric disorders across the four categories: calculated low-protein diet, estimated low-protein diet, slightly protein-reduced diet and no diet. Data include all recorded appointments (patients with psychiatric disorders: *n* = 33; patients without psychiatric disorders: *n* = 100). *p* < 0.01

Dietary adherence also differed significantly: 70% f patients without psychiatric disorders followed a calculated or estimated low-protein diet, compared to only 33% of those with psychiatric disorders (*p* < 0.01) (Fig. 6). Patients with psychiatric disorders were more likely to follow a slightly protein-reduced diet (48.5% s. 21.4%)or no protein-restricted diet (18.2% s. 8.6%).

Although there was no statistically significant difference in AAM intake frequency, patient with psychiatric disorders more often reported inconsistent or no AAM use (27.3% vs. 12.9%; *p* > 0.05) (supplementary material table S1).

Quality of life, as assessed by the *PKU-QOL* questionnaire, was significantly lower in the domains *adherence to AAM-Intake*, *practical impact on dietary protein restrictions* and the *taste of low-protein food*, *p* < 0.05 (Table 2). Patients with psychiatric disorders reported s*tomach aches* and *sadness* more frequently, whereas *slow thinking*,* aggressiveness*, and *moodiness* were reported less often (*p* < 0.05). For all other *PKU-QOL* domains, no significant difference were observed (Table S2).

**Table 2:** Differences of HRQOL between individuals with PKU with and without psychiatric disorders

Domains of PKU-QOL	with Psychiatric comorbidities	without Psychiatric comorbidities	z	*p*	*r*
	Mean ± SD MD / IQR (*n*)	Mean ± SD MD / IQR (*n*)			
Stomach aches	22.2 ± 27.2 0.0 / 50.0 (27)	7.5 ± 17.2 0.0 / 0.0 (70)	**-3.025**	**0.002**	**0.307**
Slow thinking	9.8 ± 21.9 0.0 / 18.8 (28)	18.2 ± 21.7 0.0 / 25.0 (70)	**-2.156**	**0.031**	**0.218**
Aggressiveness	8.0 ± 19.3 0.0 / 0.0 (28)	15.4 ± 22.6 0.0 / 25.0 (70)	**-1.973**	**0.048**	**0.199**
Moodiness	14.3 ± 20.9 0.0 / 25.0 (28)	27.9 ± 26.8 25.0 / 50.0 (70)	**-2.347**	**0.019**	**0.237**
Sadness	32.8 ± 26.0 25.0 / 50.0 (29)	16.4 ± 22.5 0.0 / 25.0 (70)	**-3.038**	**0.002**	**0.305**
Adherence to AAM-Intake	28.4 ± 24.9 25.0 / 41.7 (22)	17.0 ± 20.7 8.3 / 33.3 (62)	**-2.293**	**0.022**	**0.250**
Practical impact of dietary protein restrictions	35.3 ± 19.7 33.3 / 28.6 (27)	25.2 ± 18.7 21.1 / 24.1 (62)	**-2.377**	**0.017**	**0.252**
Taste of low-protein food	34.2 ± 17.1 25.0 / 25.0 (19)	24.5 ± 20.1 25.0 / 25.0 (55)	**-2.031**	**0.042**	**0.236**

Data was assessed using the PKU-QOL questionnaire. Statistical analysis was performed using the Mann-Whitney u Test (z) and effect size (r) [37]. Only significant differences are presented; for other domains, please refer to the supplementary material (Table S2)

## Discussion

Evidence regarding metabolic control, quality of life and dietary habits after transition into adult care for individuals with PKU is limited. Our data contribute to strengthening interdisciplinary care for patients with inborn errors of metabolism and may help refine treatment strategies.

Overall, metabolic control remained stable after transition, with mean Phe concentrations of 998.3 ± 290.4 µmol/l over the study period. As part of the structured transition process, patients were informed about the limited evidence for the strict Phe-value target of ≤ 600 µmol. Through shared decision-making, an individualized and realistic treatment target was defined for each patient [2]. Naturally, these individualized targets need to be reassessed in the light of new evidence or patient-specific circumstances, such as pregnancy. Most patients selected 900 µmol/l as their target, but only 38% were able to remain within their range. The highest proportion within the individual range occurred at the third annual follow-up, which coincided with the lowest mean Phe concentrations (805.9 ± 392.6 µmol/l). However, only 13 patients had completed three annual visits, which may introduce bias. Previous studies also reported difficulties maintaining target Phe levels after entering adult care. Peres et al. [10] observed a decline from 51% to 37%, though they applied a fixed and lower target of 480 µmol/l. Other studies similarly report that most adult patients fail to meet the European recommendation of ≤ 600 µmol [11, 38, 39]. A meta-analysis found an average Phe concentration of 954 µmol/l - above current recommendations but below the former 1997 threshold of 1200 µmol/l [40]. This highlights the need for stronger evidence to support current guidelines and for interventions that help patients achieve lower Phe concentration.

Only two patients (4.4%) were lost to follow-up. Loss-to-follow-up rates in other studies range from 32% to 77% for adult PKU populations, in part due to insufficient adult-specific care structures [41–43]. The comparatively low rate in our cohort may be attributable to the structured local transition program; however, the follow-up period of 4.5 years is relatively short, whereas other studies assess much longer time spans. Transitioning from pediatric to adult care is a particularly vulnerable period, especially for men [44]. Reconnecting with adult patients who were treated in the pediatric clinic before 2019 remains challenging, and the true number of patients lost to follow-up is unknown. A national database – similar to the Austrian registry - could facilitate the identification of adult PKU patients and support re-integration into specialized adult care [44].

Correlation analysis demonstrated that AAM intake und dietary protein restriction had significant effects on metabolic control. Throughout the observation period, dietary habits improved, and all patients eventually followed at least a slightly protein-reduced diet and took AAM daily. These results are broadly consistent with findings from Klimek et al. [45], who reported that 55% adhered to protein-restricted diet and another 27% followed a vegetarian or vegan diet. Other studies indicated that 18% to 36% of adult PKU patients do not follow a protein-restricted diet [13, 39, 45, 46], whereas a UK study found adherence rates as low as 32% [47].

Overall *Taste of supplements*,* Food enjoyment* and *Guilt if dietary protein restriction were not followed* had the highest *PKU-QOL* score, indicating a moderate effect on quality of life [21, 48, 49] – consistent with our findings. These results underline the need for improved PKU-specific dietary options, including greater variety of low-protein foods, enhanced palatability and more individual AAM formulations to better meet the need of adult PKU patients.

In this cohort, nearly 90% of patients had comorbidities, underscoring the need for age-appropriate expertise. Mental health disorders were most frequent, followed by micronutrient deficiencies and obesity. Studies have suggested a higher prevalence of psychiatric conditions in PKU patients compared with the general population [27, 50–52]. Compared with patients with diabetes, individuals with PKU showed higher rates of intellectual disability, autism spectrum disorders, eating disorders, sleep disorders, and personality disorders, while depression was more common in diabetes [50]. However, Burlina et al. [53] found no clear increase of psychiatric comorbidities, demonstrating the need for further research to clarify the nature and pathophysiology of these conditions.

Given the high burden of comorbidities in adults with PKU, monitoring should follow the European guideline recommendations 2017 and 2025, including plasma amino acids, vitamin B12 (or homocysteine), hemogram, MCV, ferritin, vitamin D, and kidney/bone markers (creatinine, alkaline phosphatase, calcium/creatinine excretion) [2, 54]. Routine monitoring may be expanded to include annual zinc and selenium measurement and DEXA scans every 3–5 years to improve detection of micronutrient deficiencies and bone health abnormalities.

This is one of the first questionnaire-based analyses examining quality of life in PKU patients with psychiatric comorbidities and its association with metabolic control. Patients with psychiatric disorders had significantly higher Phe concentrations, were less likely to achieve and remain within their individualized target ranges and expressed greater anxiety regarding high Phe levels. Several studies have reported associations between elevated Phe concentrations and psychiatric diagnoses [51–53]. In our cohort, *PKU-QOL* analysis revealed altered symptom patterns: patients with psychiatric conditions reported more frequent stomach pain and sadness, but less often aggressiveness, slow thinking, and moodiness. This partially contradicts the assumption that psychiatric disorders exacerbate symptom burden, potentially reflecting altered self-perception [55]. Additionally, the gut-brain-axis has received increasing attention. Gut microbiota interact with neural pathways involved in stress response, circadian regulation, and dietary metabolism [56]. Gastrointestinal symptoms correlate strongly with psychiatric disorders, especially depression and anxiety [57]. Metabolic diseases - including PKU – have been associated with altered gut microbiota, but the underlying mechanisms remain poorly understood [58]. More research is needed to elucidate potential interactions between psychiatric disorders, symptom perception, and microbiota composition in adults with PKU.

Patients with psychiatric disorders were less likely to follow a low-protein diet and were less consistent in their AAM intake. This aligns with their PKU-QoL results – showing greater impact of *adherence to AAM*, *practical impact on dietary protein restrictions* and the *taste of low-protein food*. Negative perceptions of dietary therapy may contribute to elevated Phe levels, creating a vicious circle: poor adherence leads to higher Phe concentrations, which can worsen cognitive and emotional symptoms, further impairing adherence. Over time, this may reduce both metabolic stability and mental well-being. These findings highlight the need for targeted interventions by a multidisciplinary team, including psychological support.

This study has several limitations. Although blood tests, the *PKU-QOL* und WHO-5 were assessed in a standardized manner, regular clinic visits were conducted using a uniform protocol, potentially introducing variability. Dietary habits were self-reported and not objectively verified, increasing the risk of overreporting or underreporting biases. A more structured approach - such as detailed food diaries - could improve accuracy, though routine clinical data often better represent real-world behavior. Another limitation concerns symptom evaluation. It is difficult to determine whether symptoms improved or worsened over time, as patients may have adapted to chronically elevated Phe levels and may underestimate certain symptoms - particularly those without access to ongoing PKU-specific therapy. The *PKU-QOL* may not fully capture this, possibly leading to underestimation of symptom burden.

The recently validated *PKU Symptom Severity and Impacts Scale* may help address this issue [59]. Furthermore, the number of patients at later follow-ups decreased due to insufficient follow-up duration. Finally, not all patients adhered to regular dried blood spot submissions; therefore, outpatient Phe values may not fully reflect day-to-day variability.

A key strength of this study is its use of real-world clinical data collected over a period of 4.5-years. Patients were well characterized, and the loss-to-follow-up rate was low. Importantly, this is the first evaluation of a locally implemented structured transition program.

## Conclusion

A structured transition into adult care can help individuals with PKU maintain metabolic stability and quality of life. Close monitoring of dietary habits, comorbidities, and HRQOL is essential, as patients with PKU are at increased risk for psychiatric disorders, which may further impair metabolic control. Expanding multidisciplinary care - including psychological support - may improve adherence, reduce long-term healthcare costs, and enhance overall well-being. Further research with larger cohorts and longer follow-up is warranted.

## Supplementary Information

Below is the link to the electronic supplementary material.

Supplementary Material 1

## Data Availability

The datasets supporting the conclusions of this article are included within the article and its additional files.
