# Supplementary material for: Navigating adulthood with PKU: metabolic outcomes, quality of life, and mental health 4.5 years post-transition
Source: Orphanet J Rare Dis. 2026 Jan 9;21:28. doi: 10.1186/s13023-025-04186-1 (PMC12838461; doi:10.1186/s13023-025-04186-1)
Supplement: Supplementary file 1 — Supplementary Material 1 [file 13023_2025_4186_MOESM1_ESM.docx]

# **Supplementary materials**

**Table S1:** Comparison of dietary habits of patients with and without psychiatric comorbidities.

| Dietary habits | Without psychological disorders % (n) | With diagnosed psychological disorder % (n) |
| --- | --- | --- |
| AAM intake |  |  |
| ≥ 3x/day | 67.1% (47) | 48.5% (16) |
| 2x/day | 12.9% (9) | 21.2% (7) |
| 1x/day | 7.1% (5) | 3.0% (1) |
| <1x/day | 1.4% (1) | 6.1% (2) |
| No AAM intake | 11.4% (8) | 21.2%] (7) |
| Diet |  |  |
| Calculated low-protein diet | 35.7% (25) | 6.1% (2) |
| Estimated low-protein diet | 34.3% (24) | 27.3% (9) |
| Slightly protein-reduced diet | 21.4% (15) | 48.5% (16) |
| No diet | 8.6% (6) | 18.2% (6) |

Data shown from all appointments (appointments of patients with psychiatric disorders: n = 33; appointments of patients without psychiatric disorders: n = 100). Significant difference in followed protein-low diet, p < 0.05. Differences in AAM intake were not significant, p > 0.05.

**Table S2:** Comparison of PKU-QOL domains of patients with and without psychiatric disorders.

| Domains of PKU-QOL | with Psychiatric comorbidities | without Psychiatric comorbidities | *z* | *p* | *r* |
| --- | --- | --- | --- | --- | --- |
|  | Mean ± SD  MD / IQR (n) | Mean ± SD  MD / IQR (n) |  |  |  |
| Self-rated health status | 50.0 ± 27.5 50.0 / 50.0 (29) | 41.4 ± 26.5 50.0 / 25.0 (70) | -1.394 | 0.163 | **–** |
| headaches | 26.7 ± 25.8 25.0 / 50.0 (29) | 22.1 ± 23.1 25.0 / 50.0 (70) | -0.758 | 0.448 | **–** |
| Stomach aches | **22.2 ± 27.2 0.0 / 50.0 (27)** | **7.5 ± 17.2 0.0 / 0.0 (70)** | **-3.025** | **0.002** | **0.307** |
| Tiredness | 30.2 ± 23.5 25.0 / 38.0 (29) | 27.5 ± 23.8 25.0 / 50.0 (70) | -0.537 | 0.591 | **–** |
| Lack of concentration | 29.3 ± 29.9 25.0 / 50.0 (29) | 27.9 ± 23.5 25.0 / 50.0 (70) | -0.166 | 0.868 | **–** |
| Slow thinking | **9.8 ± 21.9 0.0 / 19.0 (28)** | **18.2 ± 21.7 0.0 / 25.0 (70)** | **-2.156** | **0.031** | **0.218** |
| Trembling hands | 15.2 ± 18.4 0.0 / 25.0 (28) | 13.9 ± 23.9 0.0 / 25.0 (70) | -1.010 | 0.313 | **–** |
| Irritability | 33.0 ± 25.5 50.0 / 50.0 (28) | 37.9 ± 25.4 50.0 / 25.0 (70) | -0.744 | 0.457 | **–** |
| Aggressiveness | **8.0 ± 19.3 0.0 / 0.0 (28)** | **15.4 ± 22.6 0.0 / 25.0 (70)** | **-1.973** | **0.048** | **0.199** |
| Moodiness | **14.3 ± 20.9 0.0 / 25.0 (28)** | **27.9 ± 26.8 25.0 / 50.0 (70)** | **-2.347** | **0.019** | **0.237** |
| Sadness | **32.8 ± 26.0 25.0 / 50.0 (29)** | **16.4 ± 22.5 0.0 / 25.0 (70)** | **-3.038** | **0.002** | **0.305** |
| Anxiety | 21.6 ± 25.6 0.0 / 50.0 (29) | 13.9 ± 24.7 0.0 / 25.0 (70) | -1.616 | 0.106 | – |
| Domains of PKU-QOL | **with** Psychiatric comorbidities | **without** Psychiatric comorbidities | *z* | *p* | *r* |
|  | Mean ± SD  MD / IQR (n) | Mean ± SD  MD / IQR (n) |  |  |  |
| Adherence to supplements | **28.4 ± 24.9 25.0 / 41.7 (22)** | **17.0 ± 20.7 8.3 / 33.3 (62)** | **-2.293** | **0.022** | **0.250** |
| Guilt if poor adherence to supplements | 29.0 ± 26.7 25.0 / 50.0 (25) | 39.8 ± 26.7 25.0 / 25.0 (59) | -1.595 | 0.111 | **–** |
| Impact of supplements on family | 1.0 ± 5.1 0.0 / 0.0 (24) | 6.7 ± 16.9 0.0 / 0.0 (63) | -1.506 | 0.132 | **–** |
| Practical impact of supplements | 9.8 ± 19.0 0.0 / 12.5 (23) | 8.9 ± 17.4 0.0 / 12.5 (63) | -0.478 | 0.633 | **–** |
| Taste – supplements | 35.2 ± 19.9 50.0 / 25.0 (22) | 35.5 ± 22.9 25.0 / 25.0 (62) | -0.260 | 0.795 | **–** |
| Food temptation | 36.5 ± 27.5 37.5 / 37.5 (25) | 35.7 ± 25.2 37.5 / 37.5 (61) | -0.112 | 0.911 | **–** |
| Adherence to dietary protein restriction | 24.3 ± 22.4 19.4 / 29.6 (24) | 25.2 ± 23.6 19.4 / 30.6 (62) | -0.010 | 0.992 | **–** |
| Social impact of dietary protein restriction | **35.3 ± 19.7 33.3 / 28.6 (27)** | **25.2 ± 18.7 21.1 / 24.1 (62)** | **-2.377** | **0.017** | **0.252** |
| Social impact of dietary protein restriction | 16.6 ± 65.7 0.0 / 11.5 (28) | 11.2 ± 16.8 4.2 / 15.8 (61) | -1.809 | 0.070 | **–** |
| Overall impact of dietary protein restriction | 19.7 ± 11.9 18.5 / 13.9 (26) | 18.7 ± 16.7 15.4 / 21.2 (63) | -0.962 | 0.336 | **–** |
| Overall difficulty following dietary protein restriction | 32.7 ± 27.2 25.0 / 50.0 (26) | 32.3 ± 33.1 25.0 / 50.0 (62) | -0.342 | 0.732 | **–** |
| Guilt if dietary protein restriction not followed | 38.9 ± 26.3 50.0 / 25.0 (27) | 38.3 ± 29.3 25.0 / 25.0 (60) | -0.318 | 0.751 | **–** |
| Taste – low-protein food | **34.2 ± 17.1 25.0 / 25.0 (19)** | **24.5 ± 20.1 25.0 / 25.0 (55)** | **-2.031** | **0.042** | **0.236** |
| Food enjoyment | 20.0 ± 33.6 0.0 / 25.0 (24) | 15.9 ± 22 0.0 / 25.0 (60) | -0.342 | 0.732 | **–** |
| Domains of PKU-QOL | **with** Psychiatric comorbidities | **without** Psychiatric comorbidities | *z* | *p* | *r* |
|  | Mean ± SD  MD / IQR (n) | Mean ± SD  MD / IQR (n) |  |  |  |
| Emotional impact of PKU | 39.1 ± 20.0 35.0 / 35.0 (29) | 32.9 ± 16.6 35.0 / 27.5 (69) | -1.309 | 0.191 | **–** |
| Practical impact of PKU | 15.8 ± 13.0 14.6 / 18.8 (28) | 15.4 ± 16.4 8.3 / 25.0 (69) | -0.550 | 0.582 | **–** |
| Social impact of PKU | 18.9 ± 23.8 12.5 / 25.0 (29) | 12.0 ± 14.7 6.3 / 18.8 (69) | -0.987 | 0.323 | **–** |
| Overall impact of PKU | 26.6 ± 15.8 22.7 / 21.3 (29) | 21.4 ± 13.9 18.8 / 18.2 (69) | -1.569 | 0.117 | **–** |
| Anxiety – blood test | 9.0 ± 17.1 0.0 / 12.5 (25) | 13.6 ± 27.3 0.0 / 12.5 (67) | -0.032 | 0.975 | **–** |
| Anxiety – Phe levels | **58.6 ± 30.1 50.0 / 37.5 (29)** | **30.1 ± 23.9 25.0 / 50.0 (68)** | **-4.168** | **0.000** | **0.423** |
| Anxiety – Phe levels during pregnancy | 57.8 ± 43.5 75.0 / 100.0 (16) | 50.0 ± 32.2 50.0 / 50.0 (42) | -0.853 | 0.394 | **–** |
| Financial impact of PKU | 35.3 ± 32.4 25.0 / 50.0 (29) | 32.1 ± 28.1 25.0 / 50.0 (67) | -0.357 | 0.721 | **–** |
| Information on PKU | 28.4 ± 26.5  25.0 / 50.0 (29) | 29.8 ± 23.4  25.0 / 0.0 (68) | -0.229 | 0.818 | **–** |

Statistical analysis was performed using the Mann-Whitney u Test (z) and effect size (r). Significant results are highlighted in bold (p < 0.05)
